# Long-Term Outcomes After Arterial Switch Operation: Risk Factors and the Impact of Anatomical Complexity in a Single-Centre Cohort

**DOI:** 10.1007/s00246-025-04095-x

**Published:** 2025-11-20

**Authors:** Valentina Orioli, Lucio Careddu, Valeria Francesca Mangerini, Marianna Berardi, Gabriele Egidy Assenza, Francesco Dimitri Petridis, Marta Agulli, Sara Schirru, Gaetano Domenico Gargiulo, Andrea Donti, Emanuela Angeli

**Affiliations:** 1 Pediatric and Adult Congenital Heart Disease Cardiac Surgery Department, IRCCS, Azienda Ospedaliero-Universitaria di Bologna, Bologna, Italy; 2 Pediatric Cardiology and Adult Congenital Heart Disease Program, IRCCS, Azienda Ospedaliero-Universitaria di Bologna, Bologna, Italy; 3 Anaesthesiology and Intensive Care Unit, Cardio-Thoracic-Vascular Department, St. Orsola-Malpighi Hospital, University of Bologna, Bologna, Italy; 4 Department of Experimental, Diagnostic and Specialty Medicine, DIMES, University of Bologna, Bologna, Italy; 5 Pediatric and Adult Congenital Heart Disease Cardiac Surgery Department, S. Orsola Hospital, University of Bologna, Via Massarenti 9, 40138 Bologna, Italy

**Keywords:** Trasposition of great arteries, Anatomical complexity, Arterial switch operation, Risk factors for reoperation, Coronary artery pattern

## Abstract

The arterial switch operation (ASO) is the standard surgical intervention to correct transposition of the great arteries (TGA). The aim of this study is to describe long-term outcomes after the ASO in patients with TGA, and to explore anatomical and clinical factors potentially associated with adverse outcomes, comparing simple and complex TGA. Since December 2001 to October 2023, 233 patients underwent arterial switch operation (158 simple TGA and 75 complex TGA). Kaplan–Meier analysis assessed survival and reoperation risk, Chi-square test and T-student test have been performed in order to analyse pre-, intra- and post-operative data among the two groups. In-hospital mortality was 1.7%. Overall survival rates were 97.9%, 96.8% and 96.8% at 5, 10 and 15 years, respectively. Freedom from surgical reoperation at 15 years was 98.3% in simple TGA and 91% in complex TGA at 15 years (*p* = 0.039). Reinterventions were infrequent and mainly related to pacemaker implantation and right ventricular outflow tract obstruction. Coronary artery stenosis occurred in 5 patients (2.2%). Risk factors analysis suggested that anatomical complexity such as intramural coronary artery (*p* = 0.003), aortic arch hypoplasia (*p* = 0.004), aortic arch interruption (*p* = < 0.001) and coronary reimplantation with button technique (*p* = 0.018) are predictive factors for mortality. In addition, complex TGA (*p* = 0.022) and post-operative aortic regurgitation (*p* < 0.001) are identified as potential predictive factors for reintervention. Arterial switch operation provides excellent long-term survival and low reintervention rates in TGA. These results support tailored long-term surveillance, particularly for patients with complex anatomy or coronary anomalies.

## Introduction

Neonatal arterial switch operation (ASO) has become the most common surgical procedure to correct transposition of the great arteries (TGA) with proper anatomic features (namely absence of severe aortic or pulmonary outflow abnormalities and properly shaped aortic and pulmonary semilunar valves). Jatene and Lecompte implemented and modified this anatomical surgical correction, proving through the years the benefits compared to the physiological approach proposed by Mustard and Senning [1–3]. ASO is associated with low operative mortality and satisfactory late outcomes. In approximately 50% of patients with TGA additional anomalies are present, such as interventricular septal defect (VSD), pulmonary stenosis or aortic arch anomalies characterizing complex form of TGA [4].

Early and long-term outcomes can vary significantly between patients with simple TGA (sTGA) and those with complex TGA (cTGA), especially in term of early mortality and risk of reintervention.

Although anatomical correction ensures a long term survival rate > than 90% at 20 years, some issues are still concerning, in particular anatomical alterations of the coronary arteries [5], neo-aortic valve dysfunction, and the development of right ventricular outflow tract obstruction (RVOTO) [6]. 

This study aims to review a single centre surgical experience with the ASO, comparing early and late outcome of sTGA vs. cTGA and analysing risk factors for mortality and reintervention.

## Materials and Methods

Since December 2001 to October 2023, 233 patients underwent ASO for TGA at our centre.

A retrospective analysis was conducted and hospital records were analysed.

We divided the population into two groups according to the presence of additional cardiac anomalies. The sTGA group consists in patients with no associated cardiac defect, and the cTGA group in patients with associated cardiac defect underwent ASO correction. Patients with cTGAs who did not undergo ASO but underwent other interventions were excluded from the analysis. Double outlet right ventricle (DORV) TGA were also excluded from the analysis.

Secondarily, patients were split into subgroups according to birth weight: low birth weight (LBW) less than 2500 g, normal birth weight (NBW) and macrosomal birth weight (MBW) more than 4000 g.

All clinical, surgical and follow-up records have been analysed to collect data about the native cardiac and coronary anatomy as well as the surgical technique used for associated lesions repair and coronary translocation.

All patients underwent cardiac catheterization between 12 and 24 months after surgery to evaluate coronary artery reimplantation, according with our centre protocol on TGA. The cardiac catheterisation and angiography sheets have been analysed with regard to evidence of coronary obstruction, pulmonary stenosis and aortic re-coarctation. Clinical, electrocardiographic and echocardiographic evaluation at follow-up were studied to identify signs or symptoms related to myocardial ischaemia.

Yacoub’s classification was utilized to describe coronary artery anatomy.

### Statistical Analysis

The statistical analysis was performed using IBM SPSS Statistics version 26.0 (IBM-SPSS Inc, Armonk, NY). Continuous variables were expressed as mean ± standard deviation and percentages for discrete variables. A student T test or the Mann-Whitney U test were used to compare continuous variables. Categorical variables were compared using the 2 test. Kaplan–Meier analysis was used for evaluation of survival and the risk for re-operation. Chi-square test and T-student test have been performed in order to analyse pre-, intra- and post-operative data among the two groups. Student T test and Mann-Whinteywere used to analyse variables with normal and non-normal distribution. Cox regression was chosen for univariate prognostic analysis of long-time survival and the risk for reintervention. A value of *p* < 0.05 was considered significant.

## Results

### Patient Demographics and Perioperative Outcomes

The sTGA group consists in 158 patients and the cTGA group in 75 patients. The latter includes 55 patients with TGA and VSD, 6 patients with TGA and left ventricular outflow tract obstruction (LVOTO) and 14 patients with TGA and arch anomalies, such as aortic arch interruption, hypoplasia and aortic coarctation (Table 1).

**Table 1:** Complex TGA description

TGA + VSD, *n* (%)	55 (73.3)
TGA + LVOTO, n (%)	6 (8)
TGA + arch anomalies, n (%)	14 (18.7)
Aortic arch interruption, n (%)	3 (4)
Aortic arch hypoplasia, n (%)	3 (4)
Aortic coarctation, n (%)	8 (10.7)

Values are reported as number (percentage)

Clinical and anatomical patients’ profile is described in Table 2. Median age at surgery was 7 days (IQR: 6–10) in sTGA group and 9 days (IQR: 7–17) in cTGA group. Mean weight was 3143.2 ± 461.9 and 3436 ± 1085 g in sTGA and cTGA, respectively. Bicuspid pulmonary valve was present in 3 cases (1.9%) in sTGA and 1 (1.3%) in cTGA. Rashkind’s procedure was necessary in 129 patients (81.6%) in sTGA and 55 (73.3%) in cTGA. Type A coronary artery pattern was in 101 cases (63.9%) and 46 (61.3%) in sTGA and cTGA, respectively. In sTGA group 5 patients (3.2%) had intramural coronary artery and 1 (1.3%) in cTGA group.

**Table 2:** Population and demographics characteristics

	Simple TGA (*n* = 158)	Complex TGA (*n* = 75)	Overall (*n* = 233)	*p*
Median age at surgery (days), n (IQR)	7 (6–10)	9 (7–17)	8 (6–11)	
Weight at surgery (g)	3143.2 ± 461.9	3436. ± 1085.0	3236.0 ± 730.0	0.004
Birth weight (g)	3157.7 ± 633.8	3021.3 ± 875.2	3113.8 ± 721.3	0.178
LBW, n (%)	12 (7.6)	6 (8)	18 (8.0)	0.855
Normal weigth, n (%)	138 (87.3)	64 (85.3)	202 (86.7)	0.802
Macrosomic weight, n (%)	5 (3.1)	1 (1.3)	6 (2.6)	0.430
Gestational age (weeks)	38.8 ± 1.5	38.7 ± 1.8	38.8 ± 1.6	0.496
Pre-term, n (%)	14 (8.9)	5 (6.7)	19 (8.1)	0.724
Term, n (%)	139 (88.0)	61 (81.3)	200 (85.8)	0.524
Pos-term, n (%)	2 (1.3)	0 (0)	2 (0.8)	0.354
Bicuspid pulmonary valve, n (%)	3 (1.9)	1 (1.3)	4 (1.7)	0.756
Rashkind, n (%)	129 (81.6)	55 (73.3)	184 (79.0)	0.146
Position of the great arteries (aorta to PA relationship relation to PA				
Anterior, n (%)	107 (67.7)	35 (46.7)	142 (60.9)	0.002
Anterior and rightward, n (%)	48 (30.4)	31 (41.3)	79 (33.9)	0.309
Side by side, n (%)	3 (1.9)	9 (12)	12 (5.2)	0.003
Aorto-pulmonary mismatch, n (%)	7 (4.4)	17 (22.7)	24 (10.3)	< 0.001
Coronary artery patterns				
Type A, n (%)	101 (63.9)	46 (61.3)	147 (63.1)	0.702
Type B, n (%)	11 (7.0)	6 (8.0)	17 (7.3)	0.776
Type C, n (%)	0	0	0	
Type D, n (%)	35 (22.1)	8 (10.7)	43 (18.5)	0.035
Type E, n (%)	10 (6.3)	8 (10.7)	18 (7.7)	0.247
Inverted, n (%)	1 (0.6)	7 (9.3)	8 (3.4)	0.001
Intramural, n (%)	5 (3.2)	1 (1.3)	6 (2.6)	0.410

Values are reported as mean ± SD or number (percentage)

All six patients with TGA, VSD and LVOTO underwent primary ASO. In 4 patients the LVOTO was due to a discrete subvalvular fibrous ridge, which was resected at surgery time. In the remaining 2 patients, LVOTO was caused by hypertrophy of the conal septum, treated with a limited septal myectomy.

Intraoperative data are given in Table 3. Coronary reimplantation with button technique was performed in 14 cases (8.7%) in sTGA and 14 (18.7%) in cTGA group. Autologous patch for the pulmonary artery enlargement was utilized in 99.4% and 86.7% of cases in sTGA and cTGA, respectively. Early postoperative outcomes are reported in Table 3. Postoperative echocardiography was available for all patients, aortic regurgitation (AR) was mild/moderate in 2 (1.3%) in the sTGA group and 7 (9.3%) in the cTGA group.

**Table 3:** Operative and postoperative outcomes

	Simple TGA (*n* = 158)	Complex TGA (*n* = 75)	Overall (*n* = 233)	*p*
Cardiopulmonary Bypass Time, min	149.0 ± 37.2	176.4 ± 54.4	158 ± 45.1	**< 0.001**
Aortic Cross-Clamp Time, min	100.5 ± 17.5	121.4 ± 27.4	107 ± 23.3	**< 0.001**
Re-aortic Cross-Clamp, n (%)	1 (0.6)	1 (1.3)	2 (0.9)	0.588
Coronary reimplantation technique				
Trap door, n (%)	136 (86.1)	58 (77.3)	194 (83.3)	0.095
Button, n (%)	14 (8.7)	14 (18.7)	28 (12.0)	**0.032**
Mixed, n (%)	5 (3.2)	2 (2.7)	7 (3)	0.835
PA enlargement patch				
Autologous, n (%)	157 (99.4)	65 (86.7)	222 (95.3)	**< 0.001**
Heterologous, n (%)	1 (0.6)	4 (5.3)	5 (2.1)	**0.020**
Mixed, n (%)	0	4 (5.3)	4 (1.7)	**0.003**
Postoperative outcomes				
Intubation time (h)	43.0 ± 37.4	52.5 ± 71.3	46.0 ± 50.8	0.184
ICU stay (days)	3.7 ± 2.2	4.2 ± 4.1	3.8 ± 3.0	0.258
Delayed chest closure, n (%)	6 (3.8)	6 (8)	12 (5.2)	0.175
Cardiac tamponade, n (%)	2 (1.3)	2 (2.7)	4 (1.7)	0.442
Sepsis, n (%)	5 (3.2)	2 (2.7)	7 (3.0)	0.835
Paresis of phrenic nerve, n (%)	3 (1.9)	1 (1.3)	4 (1.7)	0.756
ECMO, n (%)	3 (1.9)	3 (4)	6 (2.6)	0.344
Post operative aortic regurgitation				
Absent or trivial, n (%)	154 (97.5)	65 (86.7)	219 (94.0)	**0.002**
Mild, n (%)	2 (1.3)	7 (9.3)	9 (3.9)	**0.002**
Post operative pulmonary regurgitation				
Absent or trivial, n (%)	152 (96.2)	67 (89.3)	219 (94.0)	0.490
Mild, n (%)	4 (2.5)	3 (4.0)	7 (3)	0.490
In-hospital mortality, n (%)	2 (1.3)	2 (2.7)	4 (1.7)	0.442

Values are reported as mean ± SD or number (percentage)

Bold values indicate statistical significant

Overall in-hospital mortality rate was 1.7%.

### Long-Term Follow-Up

Late outcomes are reported in Table 4. Mean follow-up time was 8.5 ± 6.6 years (median 6.7 years) and 100% complete. No patients died at follow-up.

**Table 4:** Follow up results

	Simple TGA (*n* = 156)	Complex TGA (*n* = 73)	Overall (*n* = 229)	*p*
Coronary stenosis, n (%)	3 (1.9)	2 (2.7)	5 (2.2)	0.688
Coronary angioplasty, n (%)	0	1 (1.4)	1 (0.4)	0.142
PA stenosis, n (%)	3 (1.9)	3 (4.1)	6 (2.6)	0.332
PA angioplasty, n (%)	3 (1.9)	3 (4.1)	6 (2.6)	0.332
Aortic re-coarctation, n (%)	0	0	0	
Aortic angioplasty, n (%)	0	1 (1.4)	1 (0.4)	0.140
Aortic Regurgitation Grade				
None, n (%)	49 (31.4)	20 (27.4)	69 (30.1)	0.497
Mild, n (%)	83 (53.2)	39 (53.4)	122 (53.3)	0.939
Moderate, n (%)	11 (7.0)	7 (9.6)	18 (7.9)	0.526
Severe, n (%)	3 (1.9)	1 (1.4)	4 (1.7)	0.756
Pulmonary Regurgitation Grade				
None, n (%)	83 (53.2)	38 (52.0)	121 (52.8)	0.790
Mild, n (%)	19 (12.2)	6 (8.2)	25 (10.9)	0.354
Moderate, n (%)	35 (22.4)	18 (24.7)	53 (23.1)	0.753
Severe, n (%)	5. (3.2)	2 (2.7)	7 (3.1)	0.835
Reoperation at Follow Up, n (%)	2 (1.3)	5 (6.8)	7 (3.1)	0.061
PM implant, n(%)	0	2 (2.7)	2 (0.9)	
RVOT surgery, n(%)	1 (0.6)	2 (2.7)	3 (1.3)	
LVOT surgery, n(%)	1 (0.6)	1 (1.4)	2 (0.9)	

Values are reported as mean ± SD or number (percentage)

**Fig. 1:**
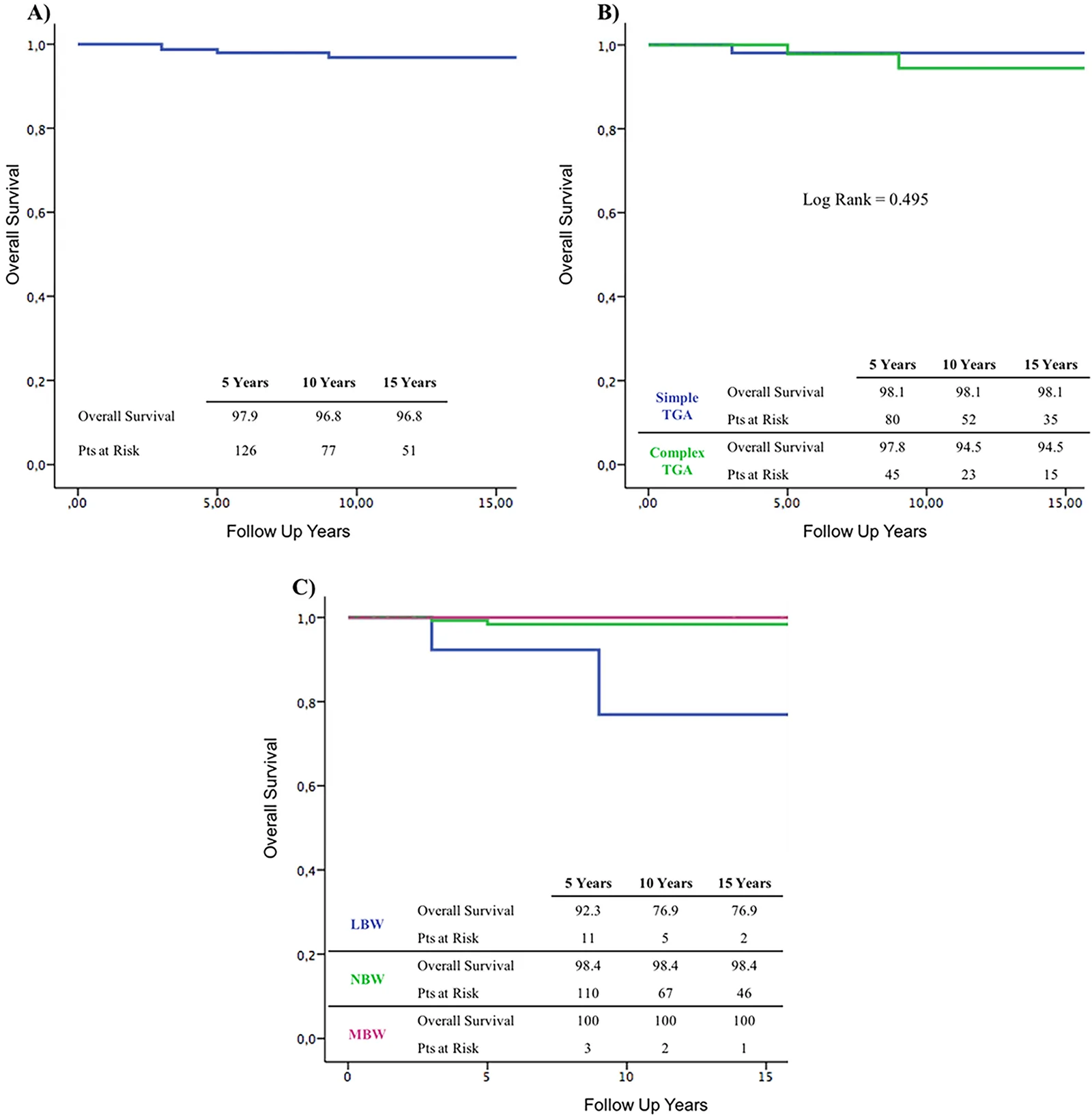
Kaplan-Meyer curves for overall survival: **A** All population, **B** comparing sTGA and cTGA, **C** comparing patients by birth weight

Kaplan-Meier curves for long-term survival is found in Fig. 1. The 5-, 10- and 15 years overall survival was 97.9%, 96.8% and 96.8%, respectively. Kaplan-Meier curve comparing the overall survival in the two groups is also reported in Fig. 1. The 5-, 10- and 15-years survival in sTGA group was, 98.1%, 98.1% and 98.1%, while in the cTGA group was 97.8%, 94.5% and 94.5%, respectively (log-rank = 0.495). In Fig. 1 we reported the Kaplan-Meier curve comparing the overall survival in LBW, NBW and MBW. The survival at 15-years in LBW group was 76.9%, in NBW group 98.4% and 100% in MBW group (Table 5).

**Table 5:** Risk factors for mortality, morbidity, coronary stenosis and reintervention

Risk factors for mortality	*P*-value	OR	95% CI
LBW	0.002	12.9	1.7–97.5
Preterm gestational age	0.003	11.8	1.6–88.8
Intramural coronary	0.004	14.9	1.3–169.7
Aortic arch hypoplasia	< 0.001	37.8	2.6–539.2
Aortic arch interruption	< 0.001	37.8	2.6–539.2
Weight at surgery	0.038		41.4–1481.9
Cardiopulmonary Bypass Time	< 0.001		− 255.8–186.5
Aortic Cross-Clamp Time	< 0.001		− 73.6 –29.1
Coronary reimplantation with button technique	0.018	7.8	1.1–57.8
Intubation time	0.014		− 112.8–13.1
Risk factors for morbidity			
LBW	0.002	5.0	1.7–14.7
Preterm gestational age	0.024	3.4	1.1–10.6
Re-aortic Cross-Clamp	< 0.001	0.1	0.7–0.1
Cardiopulmonary Bypass Time	< 0.001		− 62.1–26.8
Aortic Cross-Clamp Time	0.007		− 22.5–3.6
ICU stay	< 0.001		− 4.3–2.0
Intubation time	< 0.001		− 84.0–45.8
Risk factors for coronary stenosis			
Intramural coronary	0.005	15.1	1.3–178.4
Mixed coronary reimplantation	< 0.001	23.9	3.2–175.9
Risk factors for composite reintervention (surgical and catheter procedure)			
Complex TGA	0.022	4.0	1.1–14.0
VSD	0.013	4.4	1.2–15.6
Aortic arch hypoplasia	0.019	11.0	1.1–131.7
Aortic coarctation	0.012	6.8	1.2–37.6
Post-operative aortic regurgitation	< 0.001	13.2	2.8–62.5

Values are reported as mean ± SD or number (percentage)

*OR* odds ratio; *95% CI* 95% confidence interval

During follow-up, 6 patients required reoperation, 2 from the sTGA group and 4 from the cTGA group. In the latter, one patient underwent reoperation twice. Patients underwent reintervention for RVOTO, LVOTO and pacemaker implantation.

**Fig. 2:**
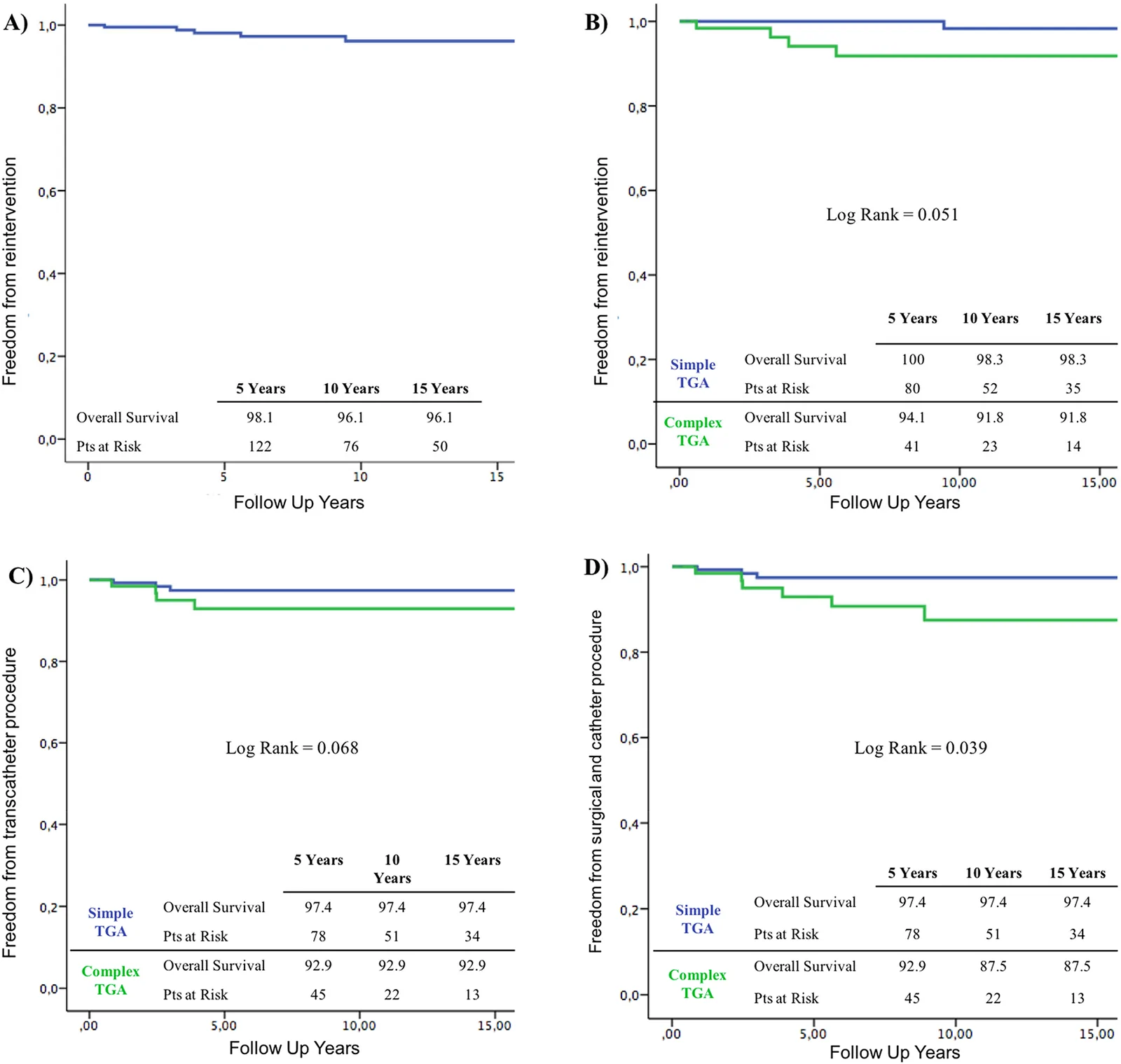
Kaplan-Meyer curves for freedom from reintervention: **A** considering the entire population, **B** comparing sTGA and cTGA. **C** Kaplan-Meyer curve for freedom from catheter reintervention comparing sTGA and cTGA. **D** Kaplan-Meyer curve for freedom from surgical and catheter reintervention comparing the two group

The overall rate for freedom from reoperation at 5, 10 and 15 years was 98.1%, 96.1% and 96.1%, respectively. Kaplan-Meier curve comparing the two groups is reported in Fig. 2. The 5-, 10- and 15-years freedom from reoperation in sTGA group was, 100%, 98.3% and 98.3%, while in the cTGA group was 94.1%, 91.8% and 91.8%, respectively (log-rank = 0.051).

Cardiac catheterization was performed in all patients at follow-up with a median time from surgery of 1.62 ± 0.8 years. In 8 patients a catheter procedure was necessary, the major indication was pulmonary artery stenosis in 6 patients (2.6%). Coronary stenosis was diagnosed in five patients. One patient underwent percutaneous coronary angioplasty. In the other four, the presence of well-developed collateral circulation supplying the occluded vessel allowed for conservative management. Two of these patients were started on beta-blocker therapy, while the others were managed with surveillance. In addition, 1 patients underwent aortic angioplasty for postoperative new-onset aortic coarctation, he was a patient with TGA and VSD who developed de novo aortic coarctation during follow-up. Freedom from transcatheter intervention, obtained with Kaplan-Meier analysis, was 97.4% in sTGA group and 92.9% in cTGA group at 15 years (log-rank = 0.068). Freedom cumulative reoperations, surgical and catheter reintervention, in sTGA was 97.4% and in cTGA 87.5% at 15 years (log-rank = 0.039). Those Kaplan-Meier curves are reported in Fig. 2.

### Risk Factors Analysis

In this study, we investigated the presence of any risk factors for mortality, morbidity, incidence of coronary stenosis and reintervention at follow-up.

Clinical patient characteristics such as LBW (*p* = 0.002), preterm gestational age (*p* = 0.003) and weight at surgery (*p* = 0.038) emerged as risk factors for mortality. In addition, anatomical variables such as intramural coronary artery (*p* = 0.004) and aortic arch interruption/hypoplasia (*p* < 0.001) showed to be risk factors for mortality. Moreover, coronary reimplantation with button technique (*p* = 0.018) has also been shown to be a risk factor for mortality. Central veno-arterial ECMO support was introduced after ASO in 6 patients (2.6%), among them 3 recovered and survived, while the remaining 3 died. ECMO emerged as predictive risk factor for mortality (*p* < 0.001).

Risk factors for morbidity emerged clinical/anatomical features such as LBW (*p* = 0.002) and preterm gestational age (*p* = 0.024).

Operative characteristics like time of cardiopulmonary bypass (*p* < 0.001), time of aortic cross clamp (*p* < 0.001) and intubation time (*p* < 0.001) were identified as risk factors for mortality and morbidity.

Intramural coronary artery (*p* = 0.005) and mixed coronary reimplantation (*p* < 0.001) were identified as risk factors for long-term coronary stenosis.

Complex TGA emerged as risk factors for reintervention, considering surgical and catheter procedure (*p* = 0.022). This is also confirmed by the isolated analysis of the presence of VSD (*p* = 0.013) and arch anomalies such as hypoplasia (*p* = 0.019) and coarctation (0.012) that were demonstrated to be risk factors for reintervention. Additionally, moderate to severe post-operative AR showed to be risk factor for reoperation (*p* < 0.001).

## Discussion

TGA is the most common congenital cyanotic heart disease at birth with a reported prevalence of 1/3500–5000 live births. ASO is the most widely used surgical intervention for TGA, achieving the anatomic correction of the defect. This procedure is crucial to restore normal circulation and ensure early and long-term survival. However, the complexity of TGA can vary significantly, with some cases presenting as sTGA and others as cTGA, which include associated anomalies such as VSD, LVOTO, and arch anomalies.

Despite advances in surgical techniques and perioperative care, the management of cTGA remains challenging. The presence of additional anatomical anomalies increases surgical’ difficulty and potentially impacts postoperative outcomes. This study aims to compare the outcomes of ASO in patients with simple versus complex TGA, with a specific focus on reintervention rates, mortality, and the identification of risk factors associated with adverse outcomes.

Our study showed that cTGAs are more associated with anatomical abnormalities such as side-by-side location of the great arteries and aorto-pulmonary mismatch further complicating the intervention. Other studies suggest a staged surgical approach in case of cTGA with anatomical features that could result in a challenging surgery [4, 7]. In our center even in case of cTGA we prefer to perform one-stage correction in neonatal period. Early repair is recommended to minimize the adverse systemic effects of prolonged cyanosis or congestive heart failure, prevent the spontaneous closure of VSDs, and reduce the risk of pulmonary vascular disease [8]. 

Otherwise, in cases of large VSD and very complex septation, palliation with banding of the pulmonary arteries prior to surgery is also provided in our center.

It has been described how in cTGA, even in presence of VSD, a Rashkind septostomy should be always considered because the quality of the ventricular mixing is often suboptimal [7, 8]. 

In our experience we noticed more aggressive approach to the Rashkind procedure over time; by now almost all patients underwent Rashkind septostomy with a median time of 12.5 ± 27.6 days before surgery without a significant difference between the groups.

### Early Outcomes

Our analysis revealed favourable short-term results. This finding corroborates previous studies that have highlighted the effectiveness of ASO in enhancing patient longevity.

Over the past four decades, advancements in ASO have led to a significant reduction in perioperative mortality rates, now falling below 5% in high-volume cardiothoracic surgical centers [9–11]. In our center the overall in-hospital mortality was 1.7%, with no difference between the groups. Early complications were also notably low. ECMO support was necessary in a low number of patients and emerged as risk factor for mortality. Kitamura et al. reported similar results in multicentre study analysing the mortality rate comparing TGA with and without VSD [7]. 

Delayed chest closure is commonly used in patients following the correction of congenital heart defects, particularly after ASO. This technique enhances stability and patient safety during the early postoperative period [4, 12, 13]. In our study, delayed chest closure was performed in 12 patients (5.2%), it was not linked to an increased rate of complications and was utilized more frequently in cases of cTGA (8%).

AR, at least moderate to severe, identified on pre-discharge echocardiogram was found to be more frequent in cTGA group (*p* = 0.002). Over time, several mechanisms have been attributed to the occurrence of AR after ASO. Among these are pre-operative aorta-pulmonary artery size discrepancy, aortic arch obstruction and presence of VSD [14, 15]. Actually, our analysis confirmed aorta-pulmonary mismatch (*p* < 0.001) and the VSD (*p* = 0.001) as risk factors for the incidence of post-operative AR, at least moderate to severe.

### Freedom from Reintervention

Reported series indicate that the need for reoperation after ASO ranges from 5% to 10% [15–19]. 

It is generally accepted that the risk of reoperation is higher in cTGA as emerged from our analysis, in fact in our series 2 patients (1.3%) in sTGA underwent reoperation, while 5 (6.8%) of the cTGA, with a median time of 7.2 ± 7.1 years from surgery. The reported operative risk connected to reoperations is low, and does not affect survival or functional outcomes [17]. 

Catheter interventions were performed in 8 patients after ASO (3.4% of survivors) with a median time of 2.1 ± 1.2 years from surgery. In the major of cases, the indication for an interventional procedure was pulmonary stenosis.

Freedom from cumulative reoperation which includes surgical and catheter procedures, is significantly lower in cTGA(*p* = 0.039). It is evident that during follow up, even in the long term, this population can develop complications requiring reinterventions more frequently than sTGA. Michalak et al. reported similar results, with a significantly lower risk of post-operative reintervention, both surgical and transcatheter in sTGA [20]. 

The most common issue following ASO is RVOTO, which requires intervention in 5–30% of cases [17]. Stenosis can occur at various levels of the RVOT. Pulmonary trunk stenosis may result from insufficient mobilization of the pulmonary arteries, leading to tension at the suture line and vessel flattening. Most frequently reported obstruction occurs at the pulmonary artery suture site, causing supravalvular stenosis of the pulmonary trunk. Disproportion between the proximal aortic segment and the pulmonary trunk, as well as inadequate growth of the pulmonary valve annulus, are also significant aetiological factors. Additionally, subvalvular or valvular stenosis are encountered [4]. 

Despite other studies [21], in our series the incidence of this complication was similar in the groups, with an overall rate of 3.9% (9 patients), of these 3 patients underwent RVOT surgery.

LVOT lesions after TGA is wildly described, but in our cohort only 2 patients (0.9%) required LVOT surgery, one patient was a sTGA who underwent Bentall procedure for dilatation of the aortic root and aortic regurgitation and the other was a cTGA with VSD who developed LVOTO. To date, reoperations for aortic valve surgery (repair or replacement) with or without ascending aorta replacement have been very rare. However, the gradual increase in significant aortic regurgitation over time, though slow, is concerning. The very long-term prognosis of the neo-aortic valve remains unknown, necessitating close observation [22, 23]. 

Lim et al. [24] reported a group of 220 patients in which aortic arch anomalies were an independent risk factor for reoperations after ASO. In our study none patients required reintervention for recoarctation; we avoid the use of enlargment patch in favour of a direct anastomosis even in complex cases with a hypoplastic or interrupted arch. The same result is reported Moll et al. [20]

In our series, coronary artery reoperations with catheter procedure for partial or total occlusion were necessary in only 1 patient (0.4%), suggesting that coronary issues have not been common during follow-up so far. None surgical reoperations for coronary artery stenosis was performed. This is in agreement with other large studies reporting a low incidence of coronary stenosis requiring reintervention [10, 22]. 

Our analysis identified as risk factors for coronary stenosis the intramural coronary artery (*p* = 0.005) and mixed coronary reimplantation (*p* < 0.001).

It has been documented that in presence of intramural coronary arteries, late coronary complications may arise after an initially uncomplicated course [25]. This is likely due to several surgical challenges encountered during the reimplantation of this type of coronary anomaly. Same consideration can be applied to mixed coronary reimplantation. We defined mixed coronary reimplantation as the application of different approaches to different coronary ostia in the same patient, reflecting the need to adapt to complex coronary anatomy. This heterogeneity may create asymmetric vectors of traction or tension on the coronary arteries, predisposing to kinking or ostial narrowing. These results can be achieved with an aggressive dissection and mobilisation of the coronary artery. Throughout the evolution of this procedure, specific problematic patterns have been identified [26]. 

### Risk Factors Analysis

In our study LBW (< 2500 g), preterm gestational age and weight at surgery were risk factors even for mortality and morbidity. Combination of LBW, prematurity, and congenital heart disease (CHD) complicates the management of these patients. Jaggers et al. analysing mortality in 3022 infants observed an increased mortality in LBW infants undergoing cardiovascular procedures for TGA [27]. Similarly, Roussin and colleagues reported an operative mortality rate of 16% in patients weighing less than 2000 g who underwent ASO [28].

Aortic arch hypoplasia or interruption emerged as significant risk factors for mortality. Patients with TGA and aortic arch obstructions are rare. Operative mortality in patients undergoing ASO combined with aortic arch repair is significantly higher than patients with sTGA, as reported by most institutions, with rates ranging widely from 1.9% to 24% [29–31]. 

Several surgical series have shown that intramural coronary arteries are associated with an increased early risk; this was clearly demonstrated in a meta-analysis published in 2002 [32]. 

Actually, this data emerged even from our analysis, where intramural coronary artery was a predictive risk factor for mortality. Moreover, coronary reimplantation with button technique has emerged as a risk factor for mortality. This technique may be chosen in specific anatomical settings (e.g., short coronary stems or widely separated ostia), or in presence of mismatch between aorta and pulmonary arteries, but it can be associated with greater risk of tension, torsion or poor orientation of the coronary ostia if coronary mobilisation is limited. These mechanical factors can increase the risk of early ischemia or difficulty haemostasis. Importantly, in our cohort the association between button technique and mortality is likely influenced by selection bias: the button technique was preferentially used in anatomically complex cases and therefore may be a surrogate for coronary or great-artery complexity rather than an independent causal factor [33].

Strong predictors for poor cumulative free-reoperation survival were cTGA, presence of VSD, aortic coarctation, aortic arch hypoplasia and moderate to severe post-operative AR. Vanini et al. in 2002 reported similar results, were cTGA, VSD, coronary anomalies and aortic coarctation were strong predictors for reoperation [15].

## Limitations

This study has several limitations. First, it is a retrospective, single-center analysis, which may limit the generalizability of the findings. Second, although the cohort size is relatively large, the number of adverse events such as mortality, coronary stenosis, and reoperations was small. As a result, the statistical power of the risk factor analysis is limited, and these associations should be regarded as exploratory rather than definitive. Finally, the study lacks external validation; multicenter or registry-based studies are needed to confirm these findings and provide stronger evidence regarding predictors of long-term outcomes after ASO.

## Conclusions

Our study provides valuable insights into the long-term outcomes of ASO, emphasizing excellent overall survival and freedom from reintervention. Overall survival was similar in both groups, but the cTGA group had higher incidence of reoperation. Exploratory analyses suggested that factors such as low birth weight, coronary anomalies, and postoperative aortic regurgitation may be associated with adverse outcomes. Our data reinforce the importance of structured long-term surveillance, particularly in patients with complex TGA or coronary anomalies, while highlighting the need for multicenter studies to validate risk stratification and optimize follow-up strategies.

## Data Availability

No datasets were generated or analysed during the current study.

## Abbreviations

ASO: Arterial switch operation
TGA: Transposition of great arteries
VSD: Ventricular septal defect
RVOTO: Right ventricular outflow tract obstruction
DORV: Double outlet right ventricle
LBW: Low birth weight
NBW: Normal birth weight
MBW: Macrosomic birth weight
LVOTO: Left ventricular outflow tract obstruction
CHD: Congenital heart disease
AR: Aortic regurgitation
